# Toward Mechanism-Directed Electrophenotype-Based Treatments for Atrial Fibrillation

**DOI:** 10.3389/fphys.2020.00987

**Published:** 2020-08-28

**Authors:** Fu Siong Ng, Balvinder S. Handa, Xinyang Li, Nicholas S. Peters

**Affiliations:** National Heart & Lung Institute, Imperial College London, London, United Kingdom

**Keywords:** atrial fibrilation (AF), fibrillation, ablation, mapping, Granger analyses

## Abstract

Current treatment approaches for persistent atrial fibrillation (AF) have a ceiling of success of around 50%. This is despite 15 years of developing adjunctive ablation strategies in addition to pulmonary vein isolation to target the underlying arrhythmogenic substrate in AF. A major shortcoming of our current approach to AF treatment is its predominantly empirical nature. This has in part been due to a lack of consensus on the mechanisms that sustain human AF. In this article, we review evidence suggesting that the previous debates on AF being *either* an organized arrhythmia with a focal driver *or* a disorganized rhythm sustained by multiple wavelets, may prove to be a false dichotomy. Instead, a range of fibrillation electrophenotypes exists along a continuous spectrum, and the predominant mechanism in an individual case is determined by the nature and extent of remodeling of the underlying substrate. We propose moving beyond the current empirical approach to AF treatment, highlight the need to prescribe AF treatments based on the underlying AF electrophenotype, and review several possible novel mapping algorithms that may be useful in discerning the AF electrophenotype to guide tailored treatments, including Granger Causality mapping.

## Introduction

Atrial fibrillation (AF) is the commonest arrhythmia, for which the direct costs alone are estimated to be up to 2.4% of the United Kingdom healthcare budget, with more than 5 million people in the United States and >8 million people in the European Union estimated to have AF in 2010 (Colilla et al., 2013; Krijthe et al., 2013). Catheter ablation currently forms the mainstay of treatment for patients with AF. Initial catheter ablation strategies were directed toward electrical isolation of the pulmonary veins, which were shown to harbor the triggers for AF (Haissaguerre et al., 1998). Pulmonary vein isolation (PVI) has therefore become a cornerstone of AF ablation, and in patients with paroxysmal AF, this has success rates of ∼70% with multiple procedures (Phlips et al., 2018; Knight et al., 2019).

The picture is more complex in persistent AF, where there are greater degrees of structural and electrophysiological remodeling (Heijman et al., 2014). AF is generally viewed as a progressive disease with “AF begetting AF” (Wijffels et al., 1995). There is evidence for significant remodeling of ionic currents, calcium handling and connexins, and structural remodeling in the form of increased fibrosis, which combine to create a pro-arrhythmic substrate in persistent AF (Nattel et al., 2008). The accepted paradigm is that, whilst triggers are relatively more important in paroxysmal AF, the substrate becomes increasingly more important in relation to the triggers with the progression to persistent AF (Heijman et al., 2014). As a result, ablation targeting only the pulmonary veins has been associated with relatively poor success rates in persistent AF (Schreiber et al., 2015).

## Adjunctive Catheter Ablation for Persistent AF

In order to address and modify the pro-arrhythmic substrate in AF, several catheter ablation strategies have been tested over the past 15 years. Linear ablations were performed to compartmentalize the atria (Jaïs et al., 2004; Hocini et al., 2005), to mirror the surgical Maze procedure (Cox et al., 2000), with the rationale that a reduction of tissue mass may increase the likelihood of AF termination, based on the critical mass hypothesis for fibrillation (Lee et al., 2013). There was also significant interest in ablating sites with complex fractionated atrial electrograms (CFAE), with the assumption that these sites represent areas of interest that are critical to driving or sustaining AF (Nademanee et al., 2004). However, there is not a clear and direct relationship between electrogram fractionation and driver sites, with fractionation also seen at sites of passive wavefront collision and “zig-zag” activation through fibrotic regions (de Bakker and Wittkampf, 2010). Despite initial interest in these adjunctive ablation approaches, the STAR-AF 2 study showed that such empirical ablation did not improve ablation efficacy, and the success rates for linear lesions or CFAE ablation in addition to PVI were similar to that of PVI alone (Verma et al., 2015).

More recently, there have been efforts to directly map and ablate the drivers of AF. These include the use of bi-atrial basket catheters to perform endocardial mapping (Narayan et al., 2012; Baykaner et al., 2018) and the use of non-invasive electrocardiographic imaging (ECGI) to perform epicardial mapping (Haissaguerre et al., 2014; Knecht et al., 2017). Although these approaches initially held great promise, subsequent follow-up studies have not been able to reproduce the high success rates first reported for rotor ablation (Buch et al., 2016). Another adjunctive ablation strategy is to target areas of atrial scar, on the premise that these areas are key components of the atrial arrhythmic substrate. Efforts have been directed toward either ablating the areas of scar (Jadidi et al., 2016) or electrically isolating these regions of scar (Kottkamp et al., 2016). Such ablation approaches have yet to be proven to be effective, and have several issues, including the problem that electrogram voltage, used as a surrogate for regions of scar, often correlates poorly with scar identified using late-enhancement MRI imaging (Qureshi et al., 2019).

None of the adjunctive ablation strategies above have reproducibly improved success rates of catheter ablation of persistent AF. Several investigators have thus focused on improving ablation lesion creation as means to improving success rates, on the basis that some of the failures can be attributed to inadequate and incomplete lesion creation. This includes developing specific indices to guide ablation, for example Ablation Index, incorporating the effects of force, time and power of ablation (Phlips et al., 2018), or new approaches to ablation, such as the “high-power, short-duration” approach to ablation (Winkle et al., 2019).

However, despite these efforts, there remains a ceiling to AF ablation success rates for persistent ablation, at around 50% (Scherr et al., 2015). A significant limitation is that current ablation strategies for persistent AF are predominantly empirical in nature, and the lack of patient-specific tailored treatments, directed toward the specific AF mechanism, places a ceiling on success rates of current treatments.

## Conflicting Evidence on the Mechanisms Sustaining AF

The electrophysiological mechanisms that sustain AF have long been disputed, with a consensus still lacking. The *anarchical* model of AF, held widely since Moe proposed the multiple-wavelet hypothesis in the 1960s (Moe et al., 1964), states that AF is sustained by multiple self-perpetuating activation wavelets propagating randomly through atrial tissue. Central to this model, supported by work from Allessie et al. (2010) and Eckstein et al. (2013), is an absence of localized sources, and would support an ablative strategy of creating globally distributed boundary lines confining and extinguishing wavelets (Jaïs et al., 2004; Hocini et al., 2005).

The apparently contrary *hierarchical* model of AF, states that AF is sustained by drivers, in the form of spiral waves or “rotors” (Krinskii, 1966; Pandit and Jalife, 2013), and is supported by work from the Jalife group in the 1990s (Davidenko et al., 1992), but despite a decade of clinical mapping experience, it was not until recently that any clinical evidence for such drivers, albeit disputed, has emerged (Narayan et al., 2012). Rotors are thought to be dynamic during AF and VF; initiating, meandering, terminating, and either re-emerging or superseded by others (Dharmaprani et al., 2019). At present, there is intense debate as to whether rotors, if they exist in humans, are predominantly stable, or short-lasting and mobile, in human AF. There has been a recent move away from the term “rotor” toward the more widely encompassing terms of “rotational driver” or “rotational activity,” to include spiral wave re-entry, leading circle re-entry and micro-reentry. High-resolution optical mapping of AF in explanted human hearts has suggested some rotational drivers in human AF may in fact be micro-reentrant in nature (Hansen et al., 2015), with such rotational drivers having predilection for areas of fibrosis (Zahid et al., 2016).

Recent human AF mapping data have challenged the rotor hypothesis, with detailed high-resolution contact electrode mapping in human atria failing to confirm the existence of stable rotational drivers (Allessie et al., 2010; Lee et al., 2014, 2015, 2017). Some investigators have suggested that AF may be maintained because of the dissociation between the endocardial and epicardial layers of human atria (Allessie et al., 2010). This endocardial-epicardial asynchrony of atrial activation gives rise to transmurally propagating waves, leading to the appearance of “new” focal waves at breakthrough sites in the opposite layer (Roney et al., 2018), and this continuous generation new fibrillation waves on both sides of the atrial wall contributes toward the stability and maintenance of AF.

It is evident that the current clinical literature on the mechanisms sustaining human AF is conflicting. Different investigators have reported a range of apparently contradictory electrophysiological mechanisms, as briefly discussed above. We previously reported that this discordance in findings may in part be explained by the different methodologies used in these studies (Roney et al., 2017; Li et al., 2019). We reported that the spatial resolution of data critically influences the interpretation of the underlying mechanism of fibrillation (Roney et al., 2017). There is a minimum spatial resolution requirement for correct identification of fibrillation mechanisms, with low-resolution data potentially leading to the false detection of non-existent rotational drivers. We also recently demonstrated that the specific analysis approach, including the pre-processing steps and parameterization of the analysis, can also significantly alter the interpretation of fibrillation data, with the lack of a standardized framework for fibrillation analysis contributing to the current conflicting evidence (Li et al., 2019). These challenges, amongst others, have thus far precluded any consensus on human AF mechanisms.

## The Fibrillation Electrophenotype Spectrum – A Unifying Hypothesis for AF Mechanisms?

**FIGURE 1:**
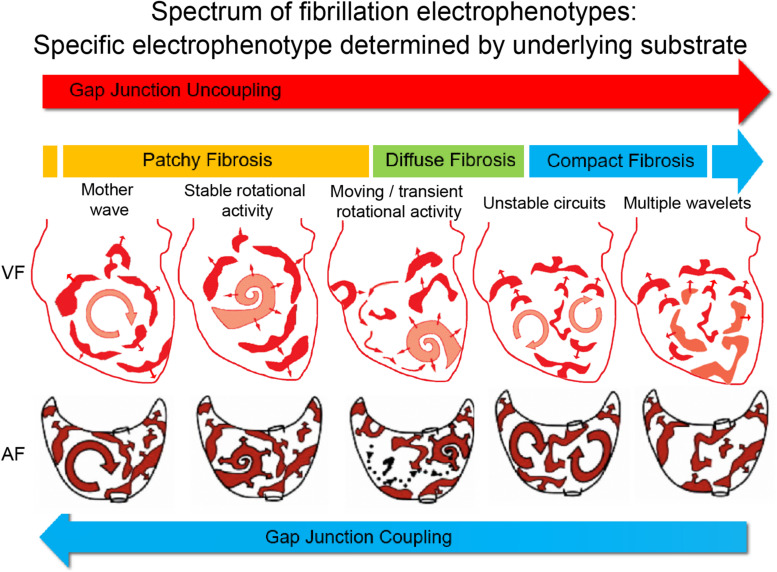
Proposed electrophenotype spectrum for myocardial fibrillation. Recent experimental data suggest that VF mechanisms exist along a continuum, ranging organized VF, sustained by focal drivers, through to disorganized VF, sustained by multiple meandering wavefronts (Handa et al., 2020b). The specific fibrillation electrophenotype is determined by the underlying substrate or electroarchitecture, with the pattern of fibrosis and gap junction coupling being two important determinants. The proposed paradigm is that AF mechanisms exist along a similar continuous spectrum, and thus treatments need to be tailored and targeted toward the specific AF electrophenotype to be successful. Figure adapted and reproduced from Eckstein et al. (2008) and Handa et al. (2020b), with permission from Oxford University Press and Elsevier.

Much of the above debate about anarchical versus hierarchical forms of AF, which raged in the early 2010s, may ultimately prove to be a false dichotomy. All the above-mentioned mechanisms may be relevant and important in sustaining AF, and the predominant AF mechanism in an individual is dependent on the specific nature of the underlying atrial substrate. We recently systematically demonstrated that a spectrum of ventricular fibrillation (VF) mechanisms exists, and that the mechanism sustaining VF is determined by the underlying substrate of the ventricular myocardium (Handa et al., 2020b). We reported a continuous range of fibrillation “electrophenoypes” that can sustain VF, influenced by the substrate spectrum that incorporates the degree of gap junction coupling and the specific pattern of fibrosis. For example, reduced gap junction coupling favors disorganized fibrillation, sustained by multiple wavelets, while preserved gap junction coupling favors organized fibrillation, sustained by rotational drivers or foci. Patchy fibrosis favors fibrillation sustained by stable rotational drivers, while compact fibrosis favors disorganized fibrillation, with diffuse interstitial fibrosis causing an intermediate electrophenotype, sustained by a combination of unstable rotational drivers and meandering wavefronts (Figure 1).

We hypothesize that a similar electrophenotype spectrum exists for AF, with the AF electrophenotype also determined by the underlying atrial substrate (Figure 1). As we have demonstrated for VF, it is possible that the degree of remodeling of the atrial substrate, including remodeling of ionic currents (Samie et al., 2001; Kneller et al., 2005; Muñoz et al., 2007), calcium handling (Samie et al., 2000), connexins, and fibrosis, determines the specific AF electrophenotype and the predominant AF mechanism. Our own analysis of human persistent AF would appear to support this hypothesis (Handa et al., 2020a).

The electrophenotype spectrum is a direct correlate of the underlying substrate spectrum, which is what determines the fibrillation mechanism. Whilst it would be desirable to accurately describe the substrate for each patient, in terms of the degree of remodeling of ionic currents, calcium handling, gap junctions, and fibrosis, that is not currently possible. However, measuring the functional correlate of the sum of the arrhythmogenic remodeling of the substrate, i.e. the electrophenotype, is within our capabilities, using the current tools and technology in the clinical electrophysiology laboratory, and thus categorising AF into broad electrophenotype categories may be a helpful concept to facilitate the tailoring of treatments.

## Mechanism-Directed Treatment Based on AF Electrophenotype

If the electrophenotype spectrum exists for human AF, then a mechanism-directed therapeutic approach, based on the AF electrophenotype, would be expected to yield better success rates than current empirical treatment strategies. For example, AF on the organized end of the electrophenotype spectrum would be more amenable to mapping and ablation of focal drivers, and responsive to Class Ic antiarrhythmic agents, which have been shown to destabilize spiral waves (Kneller et al., 2005). AF on the disorganized end of the electrophenotype spectrum may be responsive to compartmentalization of the atria or Class III antiarrhythmic agents to prolong refractoriness. Conversely, the current approach to AF treatment, which at present is neither patient-specific nor mechanism-directed, neglects the underlying electrophenotype and would be expected to have overall poor success rates.

However, such a tailored approach to AF treatment is critically dependent on the ability to accurately identify the AF electrophenotype in patients. Much of the work on AF and VF mechanisms, including our own, has been performed with high-resolution optical mapping (Davidenko et al., 1992; Muñoz et al., 2007; Roney et al., 2018; Handa et al., 2020b). The problem of low spatial resolution clinical data, which has hampered the interpretation of human AF data (King et al., 2017; Roney et al., 2017), would need to be overcome, and novel approaches for handling fibrillation data would be required.

**FIGURE 2:**
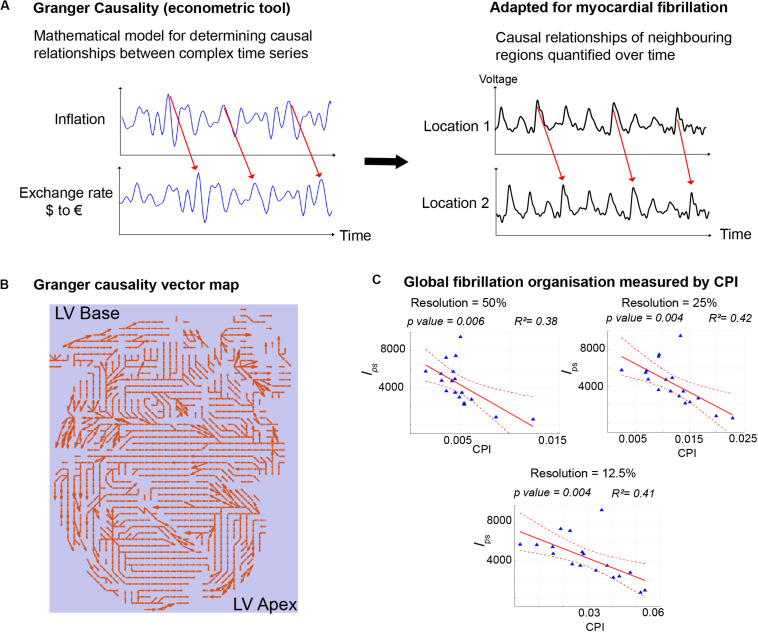
Granger Causality mapping to discern AF electrophenotype. **(A)** Granger Causality analysis, an econometric tool that determines the causal relationships between two time series, was adapted for use in fibrillation, to determine the strength of causal relationships of signals from different regions of myocardium during fibrillation. **(B)** The entire mapping field is represented with arrows depicting the strongest casual relationships. **(C)** Quantification of the number of causal pairs above a set threshold of causality (Causality Pairing Index, CPI) is a method to discern the overall organization of fibrillation and the electrophenotype of fibrillation. Importantly CPI is effective even with low resolution data, downsampled to 25%, when compared to the gold-standard high-resolution mapping data. In this example, CPI using downsampled data correlates negatively with the full-resolution l_*ps*_, which measures the number of short-lived phase singularities and is a measure of fibrillation disorganization. Figure adapted and reproduced from Handa et al. (2020a), in line with Creative Commons Attribution 4.0 (CC-BY 4.0).

There are currently several candidate approaches to identify the AF electrophenotype to guide tailored treatments. We recently adapted Granger causality analysis, originally an econometric tool for quantifying causal relationships between complex time series data (Nobel Prize Economics 2003), for use on AF data (Handa et al., 2020a). By testing for causal relationships of electrical signals between neighboring regions of the heart over time and quantifying the number of causal relationships above a set “causality threshold,” we can reliably measure global fibrillation organization and characterize the AF electrophenotype, even when applied to low spatial resolution clinical AF data (Figure 2). Other novel analysis approaches, such as wavefront field mapping, which can reveal the network of rotational and focal sites in AF (Leef et al., 2019), electrocardiographic flow mapping, which determines the main propagation patterns during AF (Bellmann et al., 2019), or mapping of connectivity between different regions of the atria usual mutual information analysis (Tao et al., 2017), may also be able to identify the AF electrophenotype to guide mechanism-directed treatments.

However, one drawback is that invasively acquired data, obtained during catheter ablation procedures, are required for these above approaches to work. The ideal scenario would be to have the ability to discern the underlying mechanism or electrophenotype of AF without the need to perform an invasive procedure, such as using data from a combination of the surface electrogram, ECGI and MRI imaging. However, these less invasive methods have their own associated limitations. Electrocardiographic imaging, has been used to map activation in both AF and VF to guide ablation of areas harboring supposed drivers, though there is ongoing debate about the degree of correlation of ECGI interpolated electrograms with those acquired with contact mapping (Cluitmans et al., 2018). Similarly, late gadolinium enhanced cardiac MRI (LGE-CMR) to detect atrial fibrosis holds promise with regards mapping the structural substrate of AF and guiding tailored therapy, though it is limited by a number of challenges in fibrosis detection posed in thin-walled atrial tissue (Siebermair et al., 2017).

## Conclusion

Current treatments for persistent AF are predominantly based on empiricism and not tailored to target the underlying AF mechanism in individual patients. Recent data suggest that fibrillation mechanisms may exist along a continuous spectrum, with the specific electrophenotype determined by the degree of remodeling of the underlying myocardial substrate and electroarchitecture. Tailored mechanism-directed treatments based on the AF electrophenotype may help to improve the currently poor success rates in treating persistent AF. Several novel mapping algorithms, including Granger Causality mapping, may be able to determine the underlying AF electrophenotype to guide tailored treatments.

## Author Contributions

FSN drafted the manuscript. All authors critically reviewed and approved the manuscript.

## Conflict of Interest

The authors have a patent application pending on Granger Causality mapping (UK Patent Application No. 1903259.8).
